# Levelling-up summer: using summer holiday programs to support child health and wellbeing – a Delphi study

**DOI:** 10.1186/s12889-025-24969-2

**Published:** 2025-11-04

**Authors:** Emily Eglitis, Timothy Olds, Rosa Virgara, Amanda Machell, Mandy Richardson, Kylie Brannelly, Carol Maher

**Affiliations:** 1 Alliance for Research in Exercise, Nutrition and Activity (ARENA), Allied Health and Human Performance, University of South Australia, North Terrace, Adelaide, SA 5000 Australia; 2 Centre for Adolescent Health, Murdoch Children’s Research Institute, Parkville, VIC Australia; 3 Deakin University, 221 Burwood Highway, Burwood, VIC 3125 Australia; 4 Department for Education, Government of South Australia, Adelaide, SA Australia; 5 National Outside School Hours Services Alliance, 66 Woodend Road, Woodend, QLD 4306 Australia

**Keywords:** Child health, Obesity, Summer holiday programs

## Abstract

**Background:**

Children’s health behaviours tend to worsen during the summer holidays, with declines in physical activity, diet, sleep, and mental wellbeing. Structured summer holiday programs may help counter these trends, while also supporting families and reducing inequalities. The purpose of this study was to establish the interest, perceived importance, and key preferences for structured summer programming in Australia and explore potential delivery models with an emphasis on sustainability, scalability and supporting families living on low incomes.

**Methods:**

A three-round Delphi study was conducted between November 2024 and April 2025. The Delphi panel consisted of stakeholders from government, extended care, research, education and parenting backgrounds. Round 1 explored perceived importance, barriers, and facilitators; Round 2 examined program features, and delivery and funding models; and Round 3 focused on priorities to improve access for families living on low incomes. Consensus was set a priori at 80%.

**Results:**

Sixty stakeholders agreed to participate, with an average response rate of 65%. There was consensus that summer programming is important for children’s physical and mental health, and social skills. Program cost, access (availability, transport) and awareness were identified as key barriers to participation. Participants agreed that programs should run for the entire working day and offer accessible, inclusive, enrichment-focused activities. For families on low incomes, providing meals and snacks was viewed as essential. A daily family contribution of $1–$10 was considered acceptable to encourage attendance and perceived value.

There was no single preferred delivery model, stakeholders emphasised the need for flexible, locally adapted approaches that build on existing community infrastructure and partnerships. Top barriers to national scale-up included lack of sustainable government funding, high operational costs, and workforce shortages. The most popular angles for advocacy were supporting children’s socio-emotional and mental wellbeing, and positive ways to spend time.

**Conclusion:**

There is clear stakeholder support for expanding structured summer holiday programming in Australia, particularly to benefit children from disadvantaged backgrounds. Programs have the potential to promote health, wellbeing, and equity, but realising this will require government investment, local partnerships, and delivery models that reflect community needs. These findings may inform similar efforts in other countries where summer programs are not equitably available.

**Supplementary Information:**

The online version contains supplementary material available at 10.1186/s12889-025-24969-2.

## Background

In many countries, the summer holidays are the longest continuous period children spend away from school. While this break allows children to unwind from the demands of the school year, it can present challenges. Research shows that many children experience academic declines over the summer [1, 2]. The absence of school also disrupts children’s routines and behaviours leading to declines in physical activity, increases in sedentary behaviour and screen time and disrupted sleep [3–7]. Dietary behaviours also tend to also worsen during the summer [8]. Consequently, children may experience accelerated weight gain and declines in fitness [9, 10]. Emerging evidence also suggests inequitable declines in mental health for some children over summer [11, 12]. This reflects the broader role of schools in promoting not just learning, but overall health and wellbeing. This protective role is linked to the structure and routine schools provide.

School days offer more structure than holidays; they involve supervised, pre-planned and segmented routines that support healthier behaviours, including more physical activity, less sedentary and screen behaviours and better sleep patterns, compared with less-structured summer days [13–16]. Schools also provide a range of other supports, such as access to sports, enrichment opportunities, meals, and social connection with peers and trusted adults. There is consistent evidence of increased summer weight gain, with some studies finding greater weight gain for at-risk groups (low socio-economic [SES], racial minority and children already overweight) [10, 17] and others finding no difference [18, 19]. More recent evidence suggests that children from lower SES backgrounds participate in less PA over summer [3, 20], but differences in diet according to SES were not evident [21].

Experiencing poverty in childhood is also linked to poorer developmental, social, cognitive and health outcomes, including higher obesity risk [22]. For families experiencing disadvantage, the absence of school can bring added financial and logistical stress, particularly when affordable childcare or youth programs are limited [23, 24]. This challenge is especially acute for single parents and those without adequate social supports, [25]. Families with greater access to financial and social resources are generally better equipped to manage the loss of school-based structure and support. Because summer represents the longest period without access to school-based support, structured programs during this time may help mitigate associated risks and summer holiday programs may offer a potential solution.

Summer programs can provide structures and supports similar to those offered by school. International evidence shows that summer programs can improve movement and diet behaviours, helping prevent accelerated summer weight gain [8, 26, 27]. In addition to supporting physical health, summer programs can also support educational outcomes and provide positive experiences that support mental, social and emotional wellbeing [11, 28, 29]. However, over three quarters of the research on children’s health and wellbeing declines over summer comes from the United States [9], with over half of the studies on the effectiveness of summer programs, also from the USA, followed by Europe [30]. Unique contextual differences of each region may limit the transferability of summer declines and effective program features to other geographical contexts. For example, Australia has relatively short summer holidays (approximately 6 weeks) and in the Southern Hemisphere, the summer holidays coincide with the Christmas period. Further, Australia has limited infrastructure for summer programs.

The aim of this study was to assess the feasibility, value and scalability of summer programs in Australia. To achieve this, we explored the experiences and perceptions of key stakeholders including parents, government representatives, educators and program providers. Stakeholders shared preferences on summer program features and delivery models, with a particular focus on supporting families living on low incomes. We used the Delphi technique to build consensus and identify key considerations for the design and delivery of summer programs, as it is a well-established method for exploring complex issues in areas with limited research evidence [31–33].

## Methods

### Study design

This study was approved by the University of South Australia Human Research Ethics Committee in accordance with the Declaration of Helsinki (protocol no. 206404). This study is reported using the CREDES framework for conducting and reporting Delphi studies (Additional file 1) [33].

### Participant eligibility and panel recruitment

Ten to 15 participants were sought from each of five diverse stakeholder groups (research, government, education, childcare and parents/guardians). Parents were included to capture lived experience of summer holiday challenges and practical considerations for program design, providing end-user perspectives to complement professional and policy viewpoints (detailed in Additional file 2). Australian stakeholders included parents of school-aged children, educators (schoolteachers or principals), extended care sector employees (educators, coordinators or advocates in public and private settings) and government personnel (state or federal employees in social, health, or education portfolios). Low SES or Indigenous individuals and communities and were not specifically targeted. International perspectives were sought from researchers in behavioural interventions for children (identified from the senior authors of original studies included in the preparatory systematic and scoping reviews [9, 28, 30]). Parent, teacher and care-sector representatives were recruited via social media posts or contacted via email through professional networks of the authorship team. Government representatives were contacted through official government websites and researchers were identified as key authors on the preparatory literature reviews. Individuals were provided with a study information sheet (background, study aims and expectations). Those who agreed to participate were emailed a link to the online survey. Participants were advised they could withdraw at any stage or participate in subsequent rounds without having completed the previous rounds. Completing the online survey was considered consent.

### Delphi survey

The Delphi technique is a well-established method for structuring group communication and, where appropriate, building consensus on complex issues [34–39]. In this study, we used the Delphi process both to seek consensus on specific program features and costs, and to explore the breadth of stakeholder perspectives on broader delivery and policy considerations. Key features of the method were retained, including anonymity of responses, iteration with controlled feedback, and statistical analysis of group ratings. A key feature of the Delphi technique is its flexible design that can be modified to align with the study’s objectives [35]. Here, considerations were made to reduce participant burden. For example, open-text responses were made available alongside multiple choice items, rank-ordering and Likert-type items. Likert items (9-point) were used to determine perceived importance (1 = not at all important, 5 = neutral, 9 = critically important) [40]. Up to four anonymous survey rounds were planned. Surveys were developed by the research team (available in Additional file 3). Surveys were conducted using Qualtrics™ software (Version XM, Qualtrics, Provo, Utah, USA). Each survey was piloted prior to use by non-participants in the Delphi proper (2 academics, 2 care-sector employees, and 2 parents). Each survey round was open for approximately 17 days, with reminder emails sent at 7, 10 and 14 days.

### Round 1

Rather than asking open-ended questions, Round 1 survey items were developed from a scoping review to establish the range of health, wellbeing and academic declines children experience over the summer holidays and systematic reviews investigating the effectiveness of summer programs in addressing physical and mental health declines over summer [9, 28, 30]. Round 1 included definitions (physical health, mental health, fitness, summer programs) and a brief conclusion of findings from the preparatory literature reviews. While the terms “school-aged child” and “children” were used throughout the three survey rounds, no age range was specified to stakeholders. From the second round onwards, each survey included a summary report of results from the previous rounds. Round 1 commenced in November 2024 and established the interest and importance of summer holiday programming in Australia. Demographic information was confirmed (stakeholder group, gender, country) and knowledge of existing local summer programming options ascertained (free-text responses were available for each item for participants to identify additional items, explain their choices or for general comments). Likert-type items were used to assess the perceived importance of summer programming overall, and specifically related to addressing academic, physical health, fitness, mental health and social declines across summer. Four multiple choice questions were used to establish barriers and facilitators to summer programming (general programs and programs to support families living on low incomes). Free-text responses were available for each item for participants to identify additional items, explain their choices or for general comments.

## Round 2

Round 2 explored program features and delivery models and commenced in January 2025. Multiple choice questions were formed based on free-text responses from Round 1. Participants were asked about optimal program structure (operation hours, availability, attendance) in the context of the Australian school year where the summer holidays run for approximately six weeks from mid-December to late January and includes the Christmas and New Year period, when many vacation programs close for 1–2 weeks. Participants were also asked to rate the importance of specific program components, overall and for programs specifically supporting families living on low incomes (9-point Likert items: 1 = not at all important, 5 = neutral, 9 = critically important) [40]. Items related to food provision, location, transport, excursions, enrichment activities, culturally inclusivity and supportive of special needs. Free-text boxes were provided to allow participants to explain their selection/rating and to ask about program accessibility (current delivery, cost and subsidies plus alternative delivery and funding models) and provide overall comments.

### Round 3

Round 3 commenced in April 2025 and clarified priority actions to enhance access to summer programming for families living on low incomes and viable delivery models for a pilot program. Items not reaching consensus in Round 2 (maximum cost to families, attendance goals and the need for transport provision) were further explored. New multiple-choice questions based on Round 2 free-text responses asked questions regarding subsidy models, methods to expand current services (delivery models, staff recruitment) and billing practices. Participants were also asked to rank-order seven key barriers to scaling summer programming and select the four most important policy priorities from a list of 12 options. Free-text responses were sought throughout the survey to explain ratings and for opportunities to scale summer programming (partnership/infrastructure and general feasibility/scalability/funding insights). Data collection was concluded after Round 3 as sufficient results were obtained.

### Data management and analysis

**Fig. 1:**
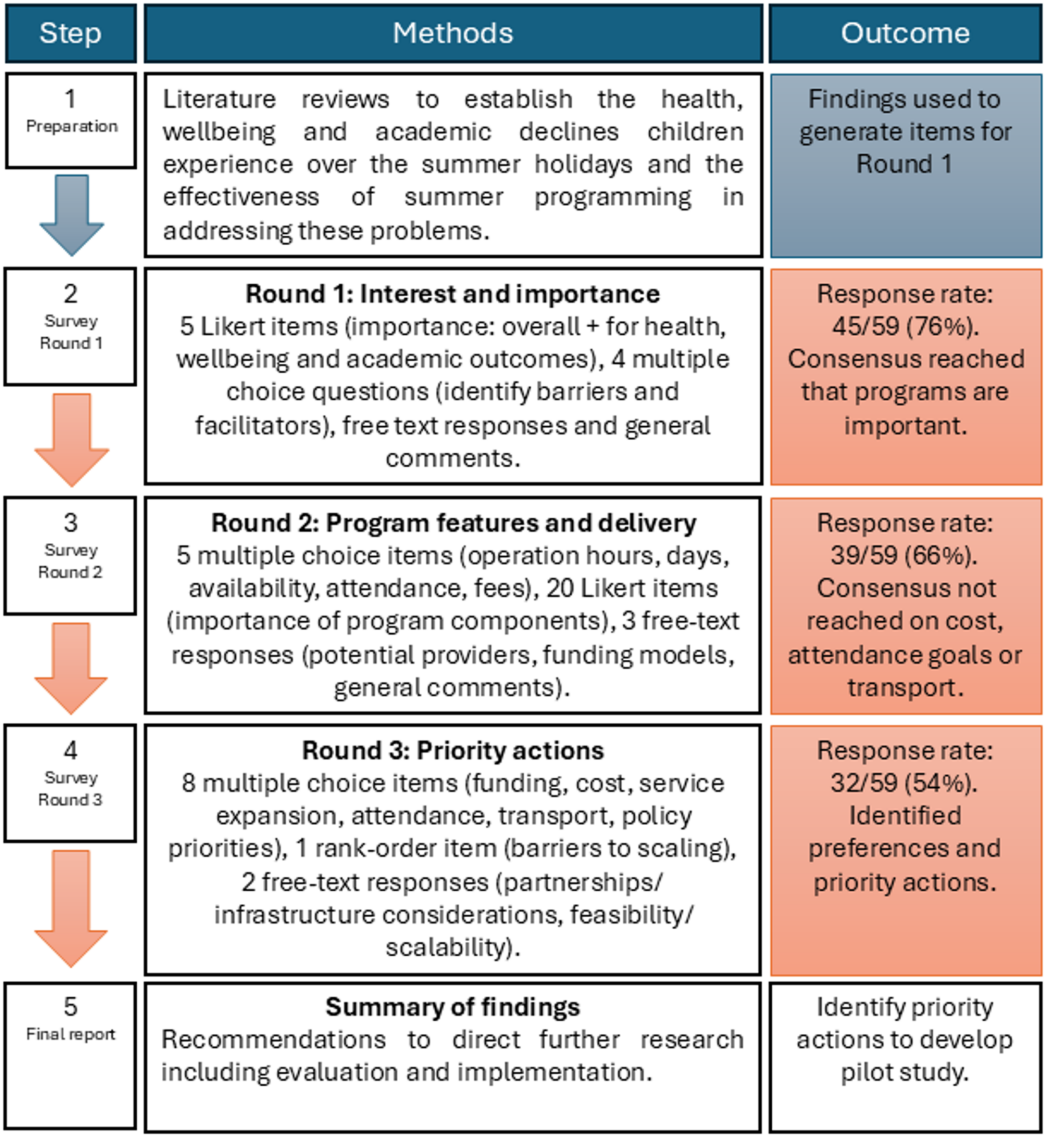
Delphi process

The response rate was calculated as the number of participants who opened the survey link, from the total number that agreed to be on the Delphi panel. Participant demographic characteristics were reported descriptively. Analysis of multiple-choice items involved identifying the most frequently selected response (mode) for each item, calculating the number of participants who selected it, and expressing this as a percentage of respondents to that item. For analysis of Likert-items, responses were collapsed into three categories (1–3 = not important, 4–6 = neutral, 7–9 = important) with consensus set as ≥ 80% of participants rating the item as important and items not reaching consensus reviewed and considered in subsequent rounds [41]. Data from individual Likert-type items are generally considered ordinal, therefore data were expressed descriptively using the mode (frequency), median (central tendency) and range (variation) [42]. Free-text responses were analysed qualitatively using inductive thematic analysis by the lead author (EE), in which codes and themes were developed directly from the data to reflect participants’ views, using NVivo software (Version 14, Lumivero, Denver, Colorado, USA). A summary of the Delphi process is provided in Fig. 1.

## Results

### Participants

The Delphi panel comprised *n* = 59 participants broadly representing both the local and international contexts. Australian perspectives (*n* = 43) were provided by parent/guardians (*n* = 10), the extended care sector representatives (*n* = 16), teacher/principals (*n* = 9) and government personnel (*n* = 8). International perspectives were provided by 16 researchers from the USA (*n* = 9), Australia (*n* = 3), and one each from Austria, Canada, Spain and the UK. The response rate for the three survey rounds was 76% (*n* = 45), 66% (*n* = 39) and 54% (*n* = 32) respectively.

### Round 1

**Fig. 2:**
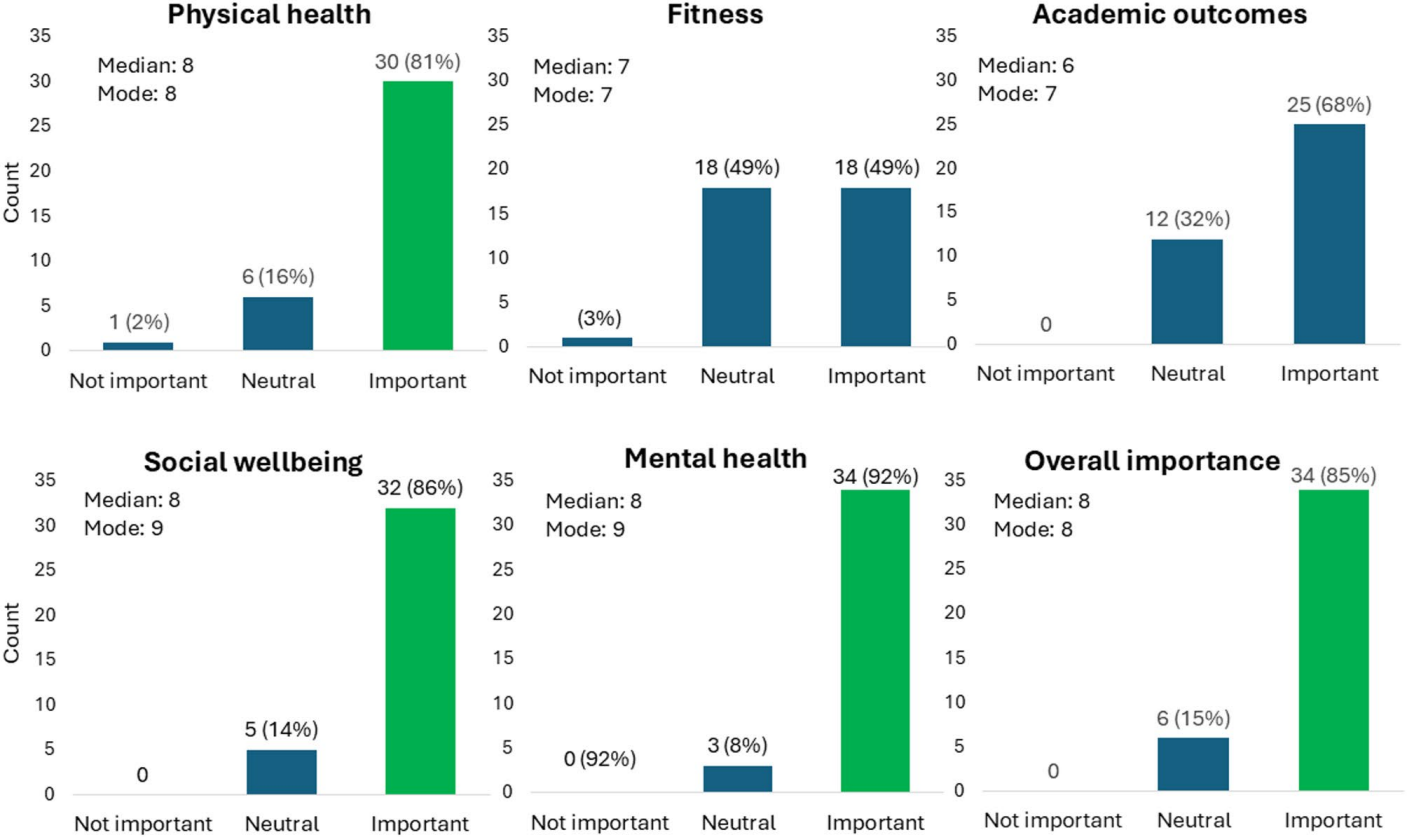
Importance of summer programs

Participants identified a variety of summer programs and formats including part-day, full-day and overnight/residential summer programs. This included library-based sessions, community-run sessions, privately delivered sports or activity sessions (e.g. soccer academy or coding academy) and vacation care services typically operated by Outside School Hours Care (OSHC) services that operate in the school holidays. Consistently, programs were based around enrichment activities like sport, activities or excursions and were perceived to support healthy behaviours (e.g. limiting screen time and increasing physical activity). Reasons that stakeholders believe summer programs are important clustered around three themes: (1) child benefits to health, wellbeing and learning; (2) parent benefits, particularly reducing stress and accessing childcare; and (3) programs address broader challenges of summer, like the need for affordable summer activities and safe spaces for vulnerable children. There was consensus that summer programming is important overall and specifically to support children’s physical health, social skills and mental health wellbeing (Fig. 2). Concerns were raised that a focus on program content before ensuring universal access could exacerbate health disparities. In addition, some participants noted that a focus on weight management might carry unintended negative consequences for children’s mental health.

A range of barriers to program engagement in general were identified including cost (97%), transport (64%) and program availability (56%). Other barriers related to program features not meeting family needs and child-specific needs. Family needs included employing well-trained staff that parents trust to manage child-specific needs (like severe allergies), operating convenient hours that consider work schedules and logistical challenges to attendance. Child-related factors included program appeal, enjoyment, age-appropriate options for older children and peer-group attendance. Facilitators to general program attendance included financial support (86%) and providing high-quality programs (61%) in convenient locations (72%). Considerate program designs are needed that encompass parent, child and community values. Programs need to engage with the community to enhance awareness, promote their benefits and increase interest. Some stakeholders reported experience with culturally diverse, regional or low SES communities (e.g., living or working in these communities) and highlighted barriers relating to misaligned program design, high-cost and poor program awareness (regarding both the existence of programs and the potential benefits of programs). Stakeholders also identified that for these communities, there are few available options and difficulty getting children to summer programs. Barriers for families on low incomes mirrored those of general programs, but with stronger agreement compared to general programs (cost; 97%, transport; 72%, availability; 64%). Several barriers also exist at the provider-level, including high operating costs and limited resources. Stakeholders identified that high cost and low availability may interact, with lack of affordability leading to low attendance, which in turn reduces the viability of running programs in those areas.

### Round 2

Round 2 identified preferences for program features and design. There was consensus that programs should operate for at least the duration of the school day (school day; 29%, or full-day; 53%). There was strong support for programs to operate throughout the summer holiday period, with the exception of only Week 2 (Christmas/New Year), which had significantly lower support. There was consensus that programs should operate in mid-late January (Weeks 3–4), suggesting that these may be core operational weeks. There was consensus that programs should be available at least five days per week (78%=5 days per week, 5%=7 days per week) with families able to choose their hours and days of attendance. With no definitive evidence on the optimal dosage of summer programming to support children’s health and wellbeing, participants shared varied views on ideal attendance frequency and duration. Some believed any attendance is beneficial, while others suggested 2–3 days per week might balance benefits of structured programming with free time. Others emphasised the importance of regular, consistent attendance to build routines, support skill development, and foster friendships.

Important program components were explored. The most common response (mode) for each component and whether or not it reached consensus is presented in Table 1. Six items achieved consensus (convenient location, enrichment activities, behaviour management strategies, culturally inclusive, support neurodiverse children, cater to children with physical disabilities) with food provision also reaching consensus for programs that support children from families on low incomes. Transport provision did not reach consensus with opinions varying considerably on this topic. Overall, transport provision was considered more important for families living on low incomes than for general programs.

**Table 1:** Importance of program components

Program component	Importance of program component (mode)	
	General summer programs	Programs supporting families living on low incomes
Provide Meals	Important (7)	*Critically important (9)*
Provide snacks	Very important (8)	*Very/Critically important (8/9)*
Convenient location	*Very important (8)*	*Critically important (9)*
Provide transport	Somewhat important (6)	Critically important (9)
Include excursions	Somewhat important (6)	Neutral (5)
Include enrichment	*Very important (8)*	*Critically important (9)*
Behaviour management	*Critically important (9)*	*Critically important (9)*
Culturally inclusive	*Important (7)*	*Important/Critically important (7/9)*
Support neurodiversity	*Important (7)*	*Critically important (9)*
Cater for physical disabilities	*Important (7)*	*Important (7)*

Items reaching consensus indicated in *italics* with modal anchor indicated for the most frequent response

In the Australian context, the identified preferences align most closely with regulated vacation care. However, these services are not universally accessible, and programs are often cost-prohibitive. Current subsidies are tied to parental workforce participation, making access particularly difficult for low-income families who are not working or studying. Therefore, these topics were explored in Round 3 along with items not achieving clear consensus in Round 2 (attendance goals, transport provision and daily costs to families).

### Round 3

Round 3 explored alternative funding and delivery models. Participants each selected two preferable options for a pilot program, and strongly supported simplified co-contribution models (60%) or government funding (53%), over expansion of existing subsidies or philanthropy/sponsorship (30% each). Participants suggested that a daily cost of $1–$10 was appropriate (73%) to enhance families’ sense of value and engagement while ensuring programs are affordable for families living on low incomes (no charge was supported by 1 participant [3%]). A small, family co-contribution was thought to promote regular attendance and enhance perceived value. Such contributions could help fund additional resources, like food. Examples of low-cost fee structures were provided, including US examples of $40 (USD) per child for six weeks of free programming with a small ($35) registration fee.

Participants supported expanding access to vacation care to meet families’ needs but acknowledged that this would bring significant challenges. Workforce shortages an ongoing concern for the care sector and impacts the quality and viability of services [43], so this was also explored in Round 3. Some respondents emphasised the importance of employing staff with specific qualifications in the care sector and while others suggested drawing on complementary professions, including youth and social workers, to help address the broader needs of children. Strategic partnerships with university programs were also proposed, for example, involving social work students completing placement hours. Additional staffing suggestions included trained volunteers with background checks, senior high school students (supervised by qualified staff), and outdoor education or adventure activity specialists.

The question of optimal attendance was explored further, but opinions remained varied. With insufficient data regarding optimal attendance to change health outcomes, it was suggested that focusing on the number of weeks attended across summer, rather than days per week, might be more practical. This approach could improve feasibility for providers in planning staffing and resources. Regarding transport, half of the respondents thought it should be provided to all children who need it while another third thought it should only be provided where access is a major barrier.

**Fig. 3:**
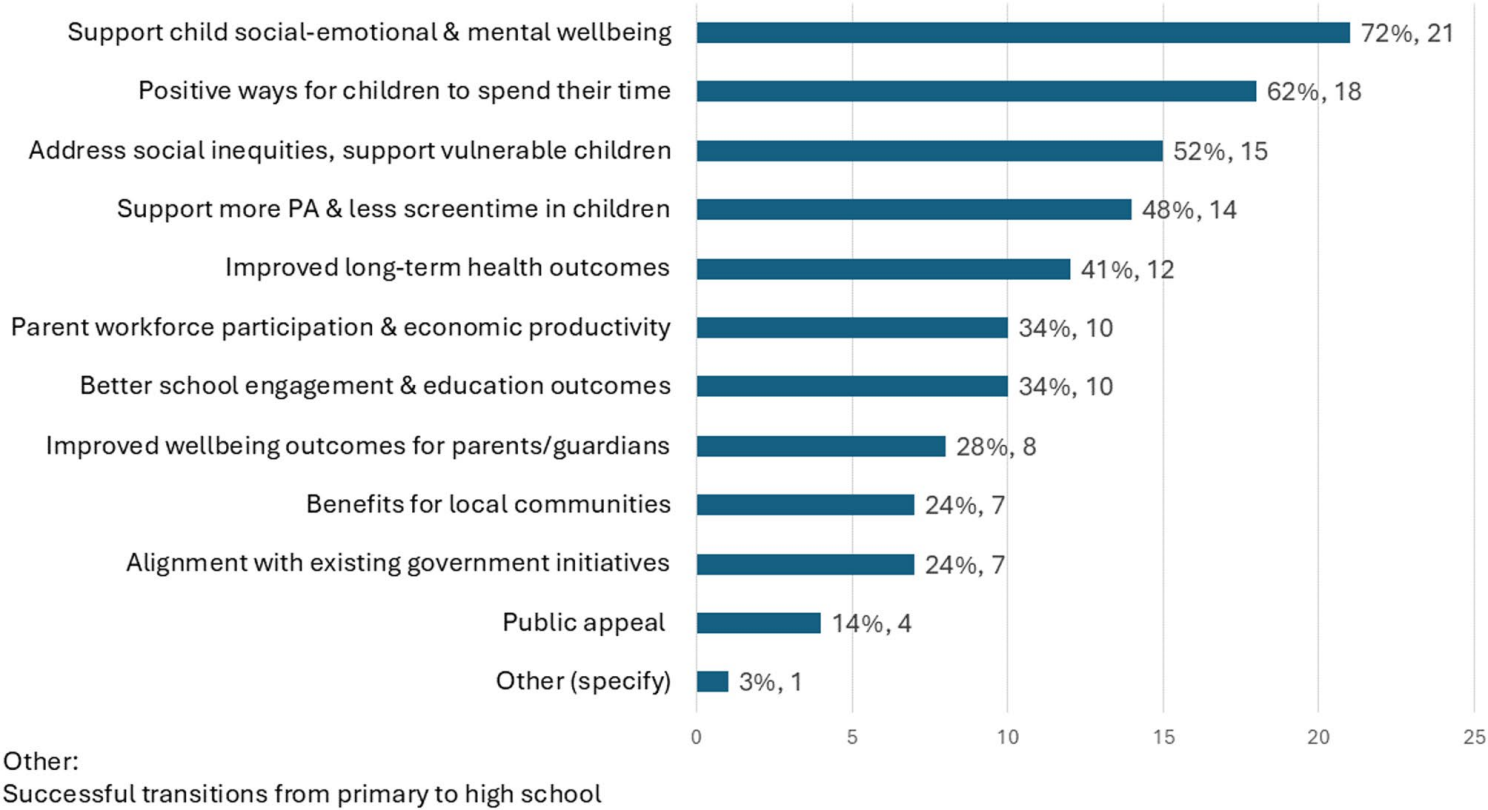
Policy positions to advocate for expansion of summer programming

The Delphi panel did not converge on a single preferred delivery model, but there was strong support exploring family-centred, context-specific approaches that prioritise universal access by leveraging existing community infrastructure and services. Key system-wide challenges to scaling programs, included lack of sustainable government funding, high operational costs and staffing shortages (Table 2). Regulatory requirements, such as the National Quality Standards and National Regulations for Vacation Care, add further cost and complexity, leading some to question whether a new sustainable delivery model specific to summer is needed. Such a model should build on existing knowledge and resource while allowing flexibility for local adaptation. It would require significant upfront investment and ongoing maintenance. To feasibly scale and fund structured summer programming three priority actions were identified: address current service gaps, leverage existing community-specific infrastructure, and build cross-sector partnerships. Stakeholders strongly supported engaging local councils, independent and government-supported providers, and community organisations to meet local needs (Table 3). Policy priorities to engage government support should focus on how summer programs support children’s social-emotional and mental wellbeing, provide positive time-use (reducing time for behaviours like delinquency and vandalism), and address social inequity by supporting vulnerable children (Fig. 3). An additional policy angle identified was summer programs assisting children transition to high school by building social skills and friendships. A summary of key findings from this Delphi study are detailed in Table 4.

**Table 2:** Barriers to scaling summer programming in Australia

Rank	Barriers ranked largest to smallest	Mode	Median
1	Lack of sustainable government funding	1 & 2	2
2	High operational costs for providers	2	2
3	Staffing shortages	3	3
4	Lack of suitable venues	5	5
5	Lack of options/expertise in delivering structured holiday programs	5	5
6	Low family engagement/uptake	6	6
7	Regulatory burden to operate under the National Quality Framework	7	6

**Table 3:** Partnerships or infrastructure to leverage for scaling summer programs

Address current challenges/gaps	Existing infrastructure	Stakeholder partnerships
• Reaching rural areas • Funding shortfalls • Reaching all “local” areas • Challenges partnering with schools	• OSHC • Community venues and infrastructure (venues that are safe, accessible and all-weather-suitable) • NFP providers • Other government programs (e.g., Dept. Agriculture) • Schools • Existing programs • Extending current offerings (e.g., “mobile holiday outreach”*)	• Enthusiasm for partnerships • Community members as “connectors” • Relationship-building with schools and principals • Local council engagement • Across multiple sectors (community, corporate, NFP)

*NFP* Not-for-profit organisations, *OSHC* Outside School Hours Care

*programs from existing service providers that can be delivered off-site at alternative locations

**Table 4:** Summary of findings

Summary finding		Stakeholder rationale
Round 1: Interest and perceived importance of structured summer programming		
1	**Summer programs in Australia**: There is a clear need and desire for additional summer programming in Australia. Stakeholders believed that it is *very important* to provide structured summer programming for all Australian children.	Summer programming has benefits for children (health, wellbeing, learning, preventing boredom), parents (childcare, address high cost of activities) and can help manage summertime challenges (consistent routines, to enrichment activities). Additional benefits exist for vulnerable children (safe, predictable spaces, contact with safe adults, healthy meals).
2	**Health**: Summer programming is *very important* in supporting physical health outcomes of Australian children (e.g., weight management, obesity prevention).	Programs support children’s health behaviours by reducing screen and sedentary time, providing opportunities for MVPA through exposure to sports participation and outside play.
3	**Wellbeing**: Summer programming is *critically important* in supporting the development of children’s social skills (e.g., maintaining peer relationships, social engagement) and supporting good mental health.	Programs provide opportunities for social interaction that can reduce isolation and support social skills and friendship building. Programs support mental health with opportunities to develop resilience, a sense of belonging and connection (socially, to community, to school), exposure to new experiences, learning and activities outside of the home.
4	**Barriers to attendance**: Low SES communities face a complex range of inter-related barriers at the systemic, social and child level. Accessibility (cost, transport, availability in local areas) are key barriers to participation that need to be addressed	Program delivery needs to address the family burden of attendance: high cost and difficulty getting children to/from programs (e.g., lack of transport and/or local service providers) reduce engagement.
5	**Awareness & appeal**: Awareness, interest and appeal for both children and families need to be considered in the design and promotion of summer programs. Opportunities exist for better community-awareness and promotion of benefits directly to children/entire peer-groups.	Parent needs/desires: childcare, programs match family values, trust in staff and providers. Child needs/desires: Engaging, fun-focussed programs, attending with friends/making friends.
6	**Barriers for program providers**: The sector faces challenges in delivering cost-efficient summer programming. Current regulatory standards, (National Quality Framework, Outside School Hours Care standards) apply to Vacation Care settings and add cost and complexity. There are added cost pressures for providers to offer inclusive programming (e.g., added staffing requirements for children on the autism spectrum).	Providers work with resource constraints (staff, facilities, funding, high operating costs) and concerns exist that current business models can prioritise profit over quality programming, fun and positive connections with children. Cost and availability may be confounding factors with lack of affordability causing low attendance which reduces the viability of running programs in those areas.
Round 2: Program feature and delivery models		
7	**Operation hours**: Summer programs should replicate the hours of a typical school day or longer. Flexible attendance hours are desirable.	These operation hours would best suit working parents and align with current operation hours for most Outside School Hours Care services.
8	**Availability**: Summer programming should be available five days per week, throughout the summer holiday period (except for the week of Christmas). The 2nd and 3rd weeks of January are likely periods of peak demand.	Consistent programming is needed throughout summer to meet diverse family needs, support families living on low incomes and best support vulnerable children. Program attendance can help families maintain beneficial routines from the school year.
9	**Program components**: For programs to support children’s health and wellbeing, it is important that they: 1. Are run in locations that are convenient for families 2. Provide enrichment activities 3. Have behaviour management strategies in place 4. Are culturally inclusive 5. Support neurodiverse children 6. Cater to children with physical disabilities To support families living on low incomes, it is *critically important* that programs also provide food (meals and snacks).	Program structure and content can impact participation but focussing on program content before universal access could exacerbate inequalities. Thoughtful design can reduce burden of participation to families (e.g., cost, location, logistics for parents) and child resistance (engaging programming, involve friendship groups, age-appropriate options 12 + years). Program components influence cost of program delivery. To enhance affordability, programs should consider alternative options for expensive components (e.g., excursions). Communities may have unique social/cultural needs to address. Food offered needs to be appropriate (e.g., appealing, non-allergenic).
10	**Transport**: Transport is a barrier to participation. There was no agreement on whether transport should be provided to all children, or only to those who experience transport as a major barrier (or at all). Program design needs to consider how families living on low incomes will get to/from the service.	For families living in lower SES communities there may be few, if any, program options available locally, with limited transport options to attend programs further away. Unreliable transport may lead to non-attendance.
Round 3: Viable delivery models, funding and priority actions for scaling and system-wide change		
11	**Expansion**: Universal access is an important first step to equitable scaling of summer programs. There is no clear preference for a single, best delivery model. A sustainable core delivery model needs to be identified that could be modified at the local level to leverage existing local knowledge and resources.	**Family-centric focus**: Cost, funding and delivery models are important factors in managing the burden of attendance (cost, transport) for families **Solve service gaps**: Current gaps in services (geographic disadvantage, options for children up to 17 years) exist where cost/funding or delivery models are inadequate **Initial and long-term planning**: There is a need to establish initial funding and partnerships and develop strategies for long-term, sustainable solutions. Opportunities exist for partnerships between government, universities, philanthropic sources, existing services and local communities.
12	**Staffing**: Some areas are experiencing a lack of “quality” trained and compassionate staff to run programs, and more staff will be required in order to expand summer program offerings. Staffing is a key challenge that could influence delivery model selection. potential solutions for recruiting extra staff included employing university students, pre-service teachers/childcare workers, community-sector staff and qualified teachers.	Well trained staff can actively cultivate trust, safety and connection for families they serve. The majority of staff should have specific skills and qualifications. Additional staff could be found in complementary professions (youth and social workers), outdoor/adventure activity-trained staff, strategic partnerships with universities (e.g., placement hours for social work students), volunteers (with appropriate background checks), year 11/12 students (under supervision of qualified staff).
13	**Attendance**: There is no clear target for optimal attendance level with summer programming. Regular, consistent attendance (2–5 days/week) is likely more optimal for health/wellbeing outcomes, forming friendships and learning new skills. Actual attendance is likely to be influenced by family factors (need for childcare, desire for child enrichment, need to balance attending programs with the benefits of free time). Families need to be able to choose their hours and days of attendance. Consider planning attendance targets around feasibility for providers (e.g. program and staffing demands) and based on total volume per summer (rather than days/week).	Respondents had diverse opinions on attendance. Some indicated that families/children will benefit from accessing a single day/week throughout summer. Consistent attendance (3–5 days/week) would likely be more optimal for improving health, but there is insufficient data regarding optimal attendance to change health outcomes. Allowing half-day attendance may improve family choice and be a lower-cost option but raises questions regarding administration, management, charging practices and if this is desirable for families. Programs run over a typical school day may need to consider pre- and post- program care for working families.
13	**Cost to families**: The approximate cost to families should be around $1–10 per day (not exceeding $15 per day). The CCSS involves complex eligibility criteria and burdensome application procedures which needs to be simplified, streamlined and out-of-pocket expenses made clearer. Where the current subsidies are inadequate (e.g., eligible services do not exist), alternative funding models are needed.	A small family co-contribution is appropriate to support sustainability of programs and may enhance engagement and perceived value of programs. There is a large administrative burden of accessing funding through the Childcare Subsidy Scheme. The process needs to be simplified with assistance provided, preferably in-person and on-site, for the application process.
14	**Alternative options**: In areas underserved by OSHC-based Vacation Care, opportunities exist for alternative delivery models. The current Childcare Subsidy is tied to specific programs/provider types so alternative funding models are needed. Funding needs equitable access (affordability, needs-based subsidies, discounts for multiple children), sustainability and family participation (payment plans and family co-contributions).	Alternative models of summer programming include things like summer day-camps. Example low-cost models from the US include $40 USD per child for six weeks or free programming with a small ($35) registration fee. Potential funding sources include dedicated government schemes (at the local, state and/or federal), expansion of existing mechanisms, fundraising or donations.
15	**Funding**: An initial investment is needed to develop a suitable delivery model prior to implementation with long-term, sustainable strategies for funding developed prior to implementation.	Significant, additional government investment is required to adequately subsize program attendance and address cost-barriers to participation.
16	**Scaling**: Addressing current service gaps is crucial for equitable scaling of summer programming and will require co-operation across sectors (levels of government, independent and government providers, community) and leveraging existing community resources.	Priority actions for scaling (Table 2): 1. Address current service gaps 2. Leverage a wide range of existing, community-specific infrastructure 3. Form stakeholder relationships across sectors: government (strong support for engaging local councils), providers (independent and government-supported) and community engagement (organisations, people, meeting community needs)

*CCSS* Childcare Subsidy Scheme, *OSHC* Outside School Hours Care, *MVPA* Moderate-vigorous physical activity

## Discussion

This Delphi study explored the feasibility and value of structured summer holiday programs for Australian children, with a focus on those from low-income households. Stakeholders – representing parents, government, education, extended care and research – reached consensus that such programs support health, wellbeing and social development and could also help families manage childcare needs during the summer. Stakeholders also emphasised that these programs could play an especially important role for disadvantaged or vulnerable children. In the absence of school, they saw summer programs as a way to provide structure, supervision, access to healthy food, and opportunities for social connection, particularly for children who might otherwise spend long periods at home without stimulation or support. For these children, programs were seen as more than just helpful; they were viewed as an essential support that could help reduce inequities and promote positive development. Participants favoured programs that run for a full day, offer fun and enriching activities, and are inclusive of all children. However, they pointed out major barriers, including cost, transport, and limited availability of programs in certain areas - particularly in lower SES and regional communities. Rather than recommending one single delivery model, participants supported flexible, local approaches that build on existing community resources. They also stressed the need for ongoing government funding to make these programs work at scale.

This study highlights the valuable role that summer programming can play in the daily lives of families, particularly those with limited resources. While some families are able to access holidays and enriching summer experiences, others face significant barriers. Summer programs can provide safe, engaging environments during a period when children are otherwise at risk of social isolation, reduced physical activity, and poor nutrition. Although summer programs may also help with childcare needs, cost remains a major barrier, particularly for low-income families who arguably stand to benefit the most from access to these services. These families experience higher rates of maternal mental health issues [22], financial stress [44], and housing insecurity [45], all of which can shape how children spend their time, creating barriers to health and development. Stakeholders recognised that in the absence of school, summer programs offer safe, structured environments that promote physical activity, support healthy weight and provide opportunities for learning and social connection [4, 13, 27, 30]. Stakeholders emphasized the need for equitable access to high-quality, child-centred care, since quality programs link to better child outcomes [46, 47].

Stakeholders identified key summer program features as including convenient locations, full-day operation aligned with school hours, and availability five-day a week throughout summer. Programs should provide food, child-centred enrichment and fun activities. In Australia, this model is similar to Vacation Care provided through Outside School Hours Care (OSHC), which covers before and after school, during holidays and on pupil-free days for children up to age 12. Additional preferences were identified that could be challenging for current vacation care models, which already face high operating expenses and staff shortages. Supporting children with special needs and challenging behaviours was seen as essential, but this requires trained staff and lowered child-to-staff ratios, something that can be difficult to achieve, especially in smaller services. Food provision was deemed important for low-income families but is not generally included in current vacation care. Providing affordable, healthy, culturally appropriate food requires careful planning. Cultural inclusivity was also emphasized. Some stakeholders specified that this could involve embedding First Nations perspectives and supporting families from non-English speaking or refugee backgrounds.

No single, delivery model was favoured. Instead, stakeholders supported flexible, locally tailored approaches that could be scaled up and adapted to different communities, while also providing options for older children. Funding and delivery models were seen as interdependent and essential to long-term sustainability. Leveraging local resources, such as school facilities and community volunteers, was suggested as ways to improve affordability and make programs relevant to local needs. Stakeholders also pointed to local government-led programs in Australia [48] and international models, including U.S. Department of Agriculture extension programs, which help reach rural areas [49]. Traditional day camps (where children attend during the day and go home each evening) and overnight residential programs are common in Europe and the U.S. These focus on recreation and enrichment and may work well for older children. However, these models are expensive [23, 50, 51], far exceeding the $10 per day that stakeholders saw as reasonable. For families with multiple children, such programs are often unaffordable, particularly for those on low incomes. While these models could complement existing vacation care, affordability and sustained access remain key challenges.

Affordability was identified as the most significant barrier to accessing summer programs. Stakeholders emphasised the need for low-cost options that limit out-of-pocket expenses for families. Suggestions included expanding existing subsidies to cover summer programs or developing new funding models designed specifically for this purpose. Interestingly, stakeholders indicated a stronger preference for a small family co-contribution over free programs. Rather than considering fees as a potential funding mechanism, a small family co-contribution (around $1–$10 per day) was seen as useful for encouraging regular attendance and giving families a sense of value - both of which would support better health and learning outcomes for children. One stakeholder believed that paying a small fee could even empower families to feel positive about their ability to provide a beneficial experience for their children. To keep costs to families low alongside high operating expenses for providers, dedicated government funding is needed.

Current subsidy schemes are tied to parents meeting work, training, or study requirements, meaning families on low incomes who are not working or studying often miss out. Additionally, the registration and application processes are complex and difficult to navigate. Stakeholders suggested simplifying qualifying criteria and offering in-person support at care sites to help families access subsidies more easily. These sentiments align with a recent review of the Australian childcare sector, which proposed removing work-activity tests and automatically providing eligibility for three subsidised care days per week to better support disadvantaged families [52]. Improving access to funding represents a demand-side strategy likely to increase participation in low-SES urban areas, where affordability limits access but demand remains high. This approach may encourage private providers, who deliver a significant proportion of Vacation Care services, to expand spaces in these areas. However, this approach may not resolve access gaps in rural, remote or culturally diverse communities, where low or inconsistent demand makes service provision financially unviable. In such settings, where community need exists but financial viability is limited, government investment in the supply side is needed to support service delivery and ensure equitable access for communities experiencing disadvantage [43, 52].

Staffing shortages were the third most cited barrier after lack of sustainable government funding and high operational costs for providers, highlighting the need for dedicated workforce planning. While some stakeholders advocated for qualified care-sector staff, others supported using professionals from related sectors, such as youth workers, social workers, educators, or outdoor activity leaders. Opportunities also exist for partnerships with training organisations and universities to support workforce development. Other cross-sector partnerships, particularly with schools and care providers, were seen as essential to ensure sustained access to facilities, staffing, and program resources.

This study has several strengths. It is the first to explore summer programming preferences in Australia, examining whether programs could improve child health and wellbeing, particularly for families on low incomes. It draws on three comprehensive literature reviews [9, 28, 30] and applies rigorous Delphi methodology, including piloted surveys and pre-defined consensus criteria. A key strength is engagement of a large and diverse Delphi panel, including representatives from government, care sector (both for-profit and not-for-profit providers), parents, teachers, and international researchers. However, several limitations warrant consideration. Delphi methodology can only reflect participating stakeholder perceptions, and these views may not capture all perspectives. While strategies were used to promote high response rates, such as personalised contact, pilot testing and reminder emails, which achieved strong initial engagement, response rates declined across successive rounds, though they remained within acceptable limits [53]. We acknowledge that some perspectives may not be fully represented in our Delphi panel. First, the voices of children were not included. While we recognise the importance of incorporating children’s views in matters that affect them, the Delphi process used in this study explored complex policy, funding, and delivery considerations that extend beyond the likely comprehension of most children, particularly in a survey format. Although the survey was not designed for an academic audience, it still required a level of literacy and conceptual understanding more suited to adult stakeholders. For these reasons, we determined that children could not meaningfully participate as Delphi panel members in this instance. Second, while parents were included as a stakeholder group, parents are an inherently diverse population and a Delphi panel of around ten cannot be expected to capture the full range of parental experiences and perspectives. This is a recognised limitation of the Delphi method, which relies on the input of a relatively small panel to reflect stakeholder views. Although this study was grounded in the Australian context, the findings are likely to be relevant in many other countries. Internationally, summer programs are often more accessible to children from well-off families, and many of the issues identified here—such as affordability, staffing, and equitable access—are common challenges worldwide. These results may be particularly useful for policymakers and practitioners in other settings looking to expand summer programming to better support child health, wellbeing and equity.

Because program delivery and funding are intricately linked, expanding summer programming will require strategic investment and policy reform. Stakeholders identified that summer programs align with broader policies promoting children’s social-emotional wellbeing, encouraging positive time-use, and supporting vulnerable children.

Future research should focus on co-designing and piloting delivery models for summer programs with local communities. Program design and evaluation should consider both efficacy and sustainability and consider incorporating important program components identified in this study. Outcome measures should include children’s academic performance, social-emotional development, and mental wellbeing. As well as considering broader family and community outcomes like parental stress, mental health, workforce participation, and parenting behaviours.

The aim of this study was to assess the feasibility, value and scalability of summer programs in Australia. Summer programs are perceived as valuable for supporting children, particularly in families on low incomes. While there is strong interest in summer programs, current delivery models have service gaps that limit feasibility and must be addressed prior to scaling. Improvements needed in current delivery models include better engagement with communities experiencing disadvantage for geographical, financial or cultural reasons and options for children over the age of 12 years. Programs need to be designed around the needs of families and funding models need to support provision of services in underserved areas to enable equitable expansion of summer programs.

## Conclusion

Summer programs are perceived as valuable for children, especially from low-income families, but equitable expansion in Australia requires closing service gaps through inclusive, family-centred design and funding. Structured summer holiday programs are a promising strategy to support children’s health and wellbeing, particularly those from low-income households. A dual investment approach, addressing family affordability and financial viability for providers, is needed to close service-gaps in disadvantaged areas. While no single, preferred delivery model emerged, stakeholders supported context-specific solutions that leverage local infrastructure and partnerships, particularly to provide options for older children. Beyond addressing childcare needs in summer, these programs could serve as prevention strategies that align with broader policy goals of supporting healthy body weight, enhancing mental wellbeing and stronger community connection. These findings provide guidance for developing equitable and scalable summer programs that support vulnerable children and families.

## Supplementary Information

Additional file 1.

Additional file 2.

Additional file 3.

## Data Availability

The datasets generated and analysed during this study are available from the corresponding author on reasonable request.

## Abbreviations

CCSS: Childcare Subsidy Scheme
MVPA: Moderate-vigorous physical activity
OSHC: Outside School Hours Care
PA: Physical activity
SES: Socio-economic status
USD: United states dollars
