# Supplementary material for: Levelling-up summer: using summer holiday programs to support child health and wellbeing – a Delphi study
Source: BMC Public Health. 2025 Nov 4;25:3782. doi: 10.1186/s12889-025-24969-2 (PMC12584409; doi:10.1186/s12889-025-24969-2)
Supplement: Supplementary file 1 — Additional file 1. [file 12889_2025_24969_MOESM1_ESM.docx]

**Additional file 2: Delphi panel recruitment strategy and inclusion criteria**

| **Stakeholder** | **Inclusion criteria** | **Recruitment Strategy**  (Target 10-15 per group) | **No. invited** | **No. agreed** | **No.**  **Participated** |
| --- | --- | --- | --- | --- | --- |
| Parent/guardian | Parent or guardian of school-aged child/children | Identified through social media  **And/or** Personal and professional contacts | 14 | 14 | 10 |
| Extended care sector:  Care provider e.g., Outside School Hours Care (OSHC), YMCA.  **Or** Australian policy or advocacy representative (e.g., National Outside School Hours Services Alliance (NOSHSA) | Individuals from local and national, public and independent care settings (vacation/OSHC): providers in a leadership role (director, coordinator), educators/practitioners  **Or** Advocacy groups: Part of organisations lobbying or advocating for children’s services or policy for childcare/extended care settings | Identified through professional contacts  **And/or** internet searches and reviews of relevant websites in the public (e.g., education.sa.gov.au, health.gov.au) and private settings  **And/or** identified through social media | 36 | 16 | 16 |
| **Teachers** or **Principals** | Individuals working as schoolteachers or principals in public or private primary or secondary schools in Australia | Identified through social media  **And/or** professional contacts | 14 | 9 | 9 |
| **Government Representatives** from any local, state or federal level of Australian government. | Individuals working in government departments related to children’s health and well-being. (e.g., health department, department of education, department of sports and recreation or other departments that are related (e.g. commission for children and young people) | Identified through professional contacts  **And/or** internet searches and reviews of relevant websites in the public (e.g., education.sa.gov.au, health.gov.au) and private settings  **And/or** identified through social media | 12 | 8 | 8 |
| **Research/Academic** in the field of behavioural interventions for children. | Researchers with international reputation for expertise in behavioural interventions for children’s health and wellbeing | Identified through professional contacts  **and/or** senior authors on key background literature e.g., systematic reviews on the effectiveness of summer programs to improve children’s health/wellbeing | 93 | 16 | 16 |
|  |  | **Total** | **168** | **63** | **59** |
