# Supplementary material for: Levelling-up summer: using summer holiday programs to support child health and wellbeing – a Delphi study
Source: BMC Public Health. 2025 Nov 4;25:3782. doi: 10.1186/s12889-025-24969-2 (PMC12584409; doi:10.1186/s12889-025-24969-2)
Supplement: Supplementary file 2 — Additional file 2. [file 12889_2025_24969_MOESM2_ESM.docx]

*Can structured summer programs improve the health and wellbeing of young Australians?*

Delphi Survey – Round 1

*Page 1 - Intro*

Thank you for participating in this Delphi survey!

# Purpose of this survey

Research shows that over the extended summer holidays, children can experience a decline in academic and health outcomes including excessive weight gain and fitness losses due to higher levels of sedentary behaviour and screen time and less physical activity. Children from families living on low incomes are affected more.

We want your ideas and opinions on how summer programs in Australia could help improve the health and wellbeing of all children.

This is the first of up to 4 surveys that will each take approximately 15 minutes to complete. Your responses are anonymous. If you would like to be involved, please click through to start. Otherwise, you may exit the survey now.

Let's get started *(survey link)*

# Section 1: Background demographics and definitions

1. **Participant Information**
2. **Please answer the following questions about yourself**

2.1 Gender

- Man
- Woman
- I identify my gender as, (please specify) (open text)
- Prefer not to say
  1. **Location: Country**
- Australia (select state)
- Outside Australia
  - specify country

1. **Stakeholder category**

**Select the stakeholder category that best represents you.** (select all that apply)

1. Parent/guardian of school aged child/children.

2. Care provider - Director, coordinator, educator or care expert from vacation care from local or national vacation care providers from the public or private setting e.g., Outside School Hours Care (OSHC), YMCA.

3. State/local government representative: Individuals responsible for relevant children’s services, education or public health. e.g., the South Australian Department for Education, Department of Human Services, Department for Sports, Recreation and Racing.

4. Federal government representative: Individuals working a department related to children’s health and well-being. e.g, Department of Health and Aged Care, Department for Education or other departments that are related (e.g. commission for children and young people).

5. Australian policy or advocacy representative: Includes individuals involved in the creation of policy for childcare/extended care settings OR  representatives of advocacy groups (organisations lobbying or advocating for children’s services and extended care) e.g., National Outside School Hours Services Alliance (NOSHSA).

6. Research/academic: In the field of behavioural interventions for children's health and wellbeing, from anywhere in the world.

If response 1 selected, next question:

- My child/children has used vacation programs
- My child/children has not yet accessed vacation care

If response 2 selected, next question:

- Public vs Private
- Job title
- Name of organisation
- Role in organisation
- Years in role

If responses 3-6 are selected, next question:

- Name of organisation
- Job title
- Years in role

If response 7 selected, next question:

- Please specify (open text)

# Definitions

Before continuing, here are some definitions of terms used:

**Good health**

For children, having “good” health means having sufficient energy, resilience, and coping skills to navigate daily challenges and reach their full potential. “Good” health enables children to participate fully in activities, learn effectively, build positive relationships, and thrive emotionally and physically.

**Physical health**

Physical health for children refers to how their bodies feel and work. Physical health includes healthy weight for age/height and adequate strength, fitness and energy to actively participate in learning and play.

*Fitness:* Fitness is a part of physical health. Here, we are referring to cardiovascular fitness, which relates to the efficiency of the heart and lungs to deliver oxygen to the muscles during physical activity, for example, in running or swimming.

**Mental health**

Mental health relates to how children feel, think, and behave every day. It's about their happiness, how they handle challenges, and how they get along with others.

**Summer programs**

Programs that are offered over the summer holidays when school is not in session. Programs can be run by private (for profit) or public (not-for-profit) organisations. Daily attendance may vary from a few hours to a full day and can be offered on one or multiple days. Examples of summer programs include vacation care, out of school hours care (OSHC) holiday programs, summer day camps.

# Section 2: Current programs

1. **Current summer program offerings**

The type and availability of summer programs varies greatly across regions. Are you aware of summer programs offered in your area?

Yes à Question 5

No/Unsure à Question 6

1. Can you describe the types of summer programs available in your area? (Open Text)
   1. Are these programs accessible to all children in your area?
      1. Yes
      2. No/unsure

**Interest and Importance**

Research shows that the summer holidays, whilst a welcome break for children, often leads to the "summer slide" i.e. a decline in their health, wellbeing, and academic skills particularly for children from families living on low-income.

Over time, this decline can increase between children from families living on low incomes and their peers. Structured summer programs have been shown to reduce this. Understanding the interest and perceived importance of such programs is crucial for developing effective and inclusive initiatives that support all Australian children.

1. **Importance:** Based on your experience and perspective, on a scale of 1 to 9, how important do you think it is to offer organised summer holiday programming for Australian children?

1. Not at all important

2. Very unimportant

3. Unimportant

4. Somewhat unimportant

5. Neutral

6. Somewhat important

7. Important

8. Very important

9. Critically important

Please explain your rating and provide any specific reasons why you believe organised summer programming is important or not important for Australian children *(Open Text)*

1. **Importance for health and wellbeing:**

On a scale of 1 to 9, how important do you think it is for summer programs to address the following areas of decline during the summer holidays? (1 = Not at all important, 9 = Critically important)

Physical health (e.g., weight management, obesity prevention)

Fitness levels (e.g., maintaining or improving cardiorespiratory fitness)

Academic skills (e.g., preventing declines in reading and math skills)

Social skills (e.g., maintaining peer relationships, social engagement)

Mental wellbeing (e.g., promoting mental health)

1. Not at all important
2. Very unimportant
3. Not important
4. Somewhat unimportant
5. Neutral
6. Somewhat important
7. Important
8. Very important
9. Critically important

Please explain your ratings. Why do you think it is (or isn't) important for summer programs to address these specific areas? *(optional, open-ended item)*

# Section 3: Barriers and facilitators

Not all children attend summer holiday programs. It is important to understand the barriers and facilitators for attendance.

1. What do you perceive as the main **barriers** to children’s participation in organised summer programs? (Select all that apply)

- Cost
- Transportation
- Awareness of programs
- Availability of programs
- Cultural or social factors
- Child’s interest/motivation
- Other (please specify)

Please provide any additional details or examples related to the barriers you selected *(optional, open-ended item)*

1. What do you perceive as the main **facilitators** to children’s participation in organised summer programs? (Select all that apply)

- Subsidies or financial support
- Convenient locations
- High-quality program offerings
- Strong marketing and outreach
- Community partnerships
- Other (please specify) *(open text)*

Please provide any additional details or examples related to the facilitators you selected *(optional, open-ended item)*

**Disadvantage**

Research has confirmed that children from families living on low income often experience stronger patterns of declines in health outcomes, health behaviours and academic achievement over summer than children from families with higher incomes.

1. What do you think are the main barriers to access for children from families living on low income in current summer programs? (Select all that apply)

- Cost
- Transportation
- Lack of awareness
- Limited availability in poor areas
- Cultural or social factors
- Other (please specify)

Please provide any additional details or examples related to the barriers you selected *(optional, open text)*

1. What measures do you think could improve the accessibility of summer programs for children from families living on low income? (Select all that apply)

- Subsidized or free program fees
- Providing transportation options
- Enhanced outreach and marketing efforts
- Partnerships with community organizations
- Programs specifically designed for poorer communities
- Other (please specify)

Please provide any additional comments or suggestions regarding the current summer program offerings and their accessibility for children from families living on low income *(optional, open text)*

1. **Other**

Do you have any other comments you’d like to add? For example, regarding important parameters (delivery models, dosage)? Or something else? *(optional, open text)*

*Thank you for your participation in this study. Your insights are valuable, and we look forward to sharing the collated results with you in the next few weeks along with the next survey round. We appreciate your continued involvement.*

Delphi Survey – Round 2

Thank you for participating in Round 2 of our Delphi study on structured summer programs for Australian children. In Round 1, stakeholders identified key barriers, facilitators, and priorities for these programs, focusing on their importance in supporting children’s physical, mental, and social-emotional wellbeing. Key findings are available (link)

**This round aims to refine those findings by exploring stakeholders’ preferences for program delivery, including settings, providers, components, and schedules.**

# Background

## Why summer programs matter

**Physical Health:** Research shows summer programs can promote healthier behaviours by providing structured opportunities for physical activity and access to nutritious food. Programs also help regulate routines, reducing sedentary time and improving sleep patterns, which benefit children’s overall health.

**Social-Emotional Wellbeing:** Summer programs foster social skills and emotional resilience through peer interactions, enrichment activities, and supportive adult mentorship. These experiences help children build a sense of belonging, self-mastery, and confidence.

**Challenges for Low-Income Families:** Families on low incomes often face barriers like affordability, accessibility, and availability, which limit children’s access to these benefits. Addressing these barriers is critical to reducing health inequities and ensuring all children can thrive during the summer break.

### Focus of this round

This survey seeks your input to refine the parameters for structured summer programs that are accessible, impactful, and sustainable, especially for families experiencing disadvantage. Your insights will help shape evidence-based recommendations to guide the design of these programs in Australia. Specifically, we aim to explore how often programs should be available, the minimum attendance required to achieve meaningful benefits, and the most suitable timing to meet families’ needs during the summer holiday period.

# Part 1

## Program structure

***Survey question 1:*** *When considering the daily operational hours for structured summer programs, which option do you think would best meet the needs of children and families?* (select one)

1. Short session (e.g. 1-2 hour session)
2. Half-day programs (i.e. 8.30am – 12.30pm)
3. School-day length programs (i.e. 8.30am – 3.30pm)
4. Long-day programs that align with typical work hours (i.e. 7.30am – 6pm)
5. Something else (specify)

***Survey question 2:*** *Please provide any other comments you have on operation hours.*

***Survey question 3:***

If we were to design a vacation care program aimed at supporting children' s mental, social, and physical health of children during the summer holidays, which weeks should the program ideally operate? Please consider both the needs of families and the feasibility of operating programs during specific weeks (e.g., availability of staff, holiday participation patterns). (Select all that apply)

**Duration Options:**

- Week 1: Week before Christmas (e.g., 18-24 December)
- Week 2: Christmas and New Year week (e.g., 25 December - 1 January)
- Week 3: First week of January (e.g., 2-8 January)
- Week 4: Second week of January (e.g., 9-15 January)
- Week 5: Third week of January (e.g., 16-22 January)
- Week 6: Fourth week of January (e.g., 23-29 January, week leading up to the start of school in some states)
- Week 7: Final week (e.g., 30 January - 4 February, week leading up to the start of school in the remaining states)
- Other (please specify): ______________________

***Survey question 4:*** *Please briefly explain why you chose that response* (open text)

## Attendance

Summer programs can play a vital role in supporting children's mental, social, and physical health during the long summer holidays. When designing a program to support children and families experiencing disadvantage, it is important to consider:

- The **minimum total attendance** required over the summer holiday period (approximately 30-35 weekdays) to achieve meaningful benefits for children.
- How often the program should be available to meet families’ needs.

**Survey question 5: Minimum attendance for benefits**
The Australian summer school holiday period typically includes around 30-35 weekdays (excluding public holidays). In your opinion, what is the minimum number of weekdays a child should attend the program over this period to achieve meaningful mental, social, and physical health benefits? (Select one)

- Fewer than 5 days
- 5–9 days
- 10–14 days
- 15–19 days
- 20–24 days
- 25 days or more
- Other (please specify): ___________

**Survey question 6: Program availability**To best support families and caregivers, how many days per week should the summer program be available? (Select one)

- 1 day per week
- 2 days per week
- 3 days per week
- 4 days per week
- All weekdays (Monday to Friday)
- Other (please specify): ___________

**Survey question 7: Attendance (open text)**

Please provide any further reasons or clarifying comments regarding attendance and availability open text).

# Part 2: Program components

Summer programs can serve a variety of needs, from supporting the health and wellbeing of all children to addressing the unique challenges faced by children from low-income families. This section seeks your input on the importance of various program features in achieving these goals.

***Survey question 8:***

Considering the components of summer programs, how important are the following features?

*(Rate each feature on a scale from 1 to 9, where 1 = Not at all important, 2 = Very unimportant, 3. Unimportant, 4. Somewhat unimportant, 5. Neutral, 6. Somewhat important, 7. Important, 8. Very important, 9. Critically important)*

Part 1: Supporting the health and wellbeing of the general population of children?

1. Provide food (meals, snacks)
2. Being located in the local area
3. Offering transport (e.g., bus service between home and the program)
4. Providing excursions
5. Providing enrichment activities (eg. Arts, sports)
6. Implementing strategies to manage challenging behaviours.
7. Offering culturally inclusive programming
8. Supporting neurodiverse children
9. Supporting children with physical disabilities.

***Survey Question 9***

Part 2: Supporting children from families living on low incomes?

1. Provide food (meals, snacks)
2. Being Located in the local area
3. Offering transport (e.g., bus service between home and the program)
4. Providing excursions
5. Providing enrichment activities
6. Implementing strategies to manage challenging behaviours.
7. Offering culturally inclusive programming
8. Supporting neurodiverse children
9. Supporting children with physical disabilities.

***Survey question 10:*** Please provide reasons for your answers.

If unsure, you might like to explain why you think certain components are important, or explain if your answers differ for programs targeting the health of the general population of children versus families living on low incomes.

# Part 3: Accessibility

Ensuring summer programs are accessible to all families, particularly those on low incomes, is essential. However, the cost of care can pose significant challenges for both providers and families. The Childcare Subsidy Scheme in Australia aims to alleviate some of these pressures but has limitations that may prevent many families from accessing its benefits.

### Current barriers to the Childcare Subsidy Scheme

While the scheme can cover up to 90% of daily fees for Out of School Hours Care (OSHC) and Vacation Care, several barriers remain:

- **Eligibility Requirements**: Families must meet strict productivity criteria (e.g., hours of work or study).
- **Means Testing**: Subsidies decrease on a sliding scale depending on family income, with adjustments made to account for additional children.
- **Complex Application Processes**: Lengthy and complicated application procedures deter many families.
- **Availability**: Some regions lack OSHC/Vacation Care facilities, making the subsidy inapplicable.

#### Fees covered by the Childcare Subsidy Scheme

***Survey question 11:*** In your opinion, what is the maximum fee (after subsidy) that families on low incomes are likely to pay per day for their child to attend a summer program? *(select one)*

- No charge
- $0-$5
- $5-10
- $10-15
- $15-$20
- $20-$25
- $25-30
- $35-40
- $40-45
- $45-50
- $50-$55
- $55-60
- $60-65
- $65-$70
- $70-$75
- $75+

#### Access to Childcare Subsidy Scheme

**Survey question 12:**

The current Childcare Subsidy Scheme provides fee subsidies for eligible families, but many families, particularly those who are vulnerable or living on low incomes, may still face barriers to accessing it.

What adjustments or modifications could be made to the Childcare Subsidy Scheme to better support families who would benefit from summer programs but are not currently accessing the subsidy?

*Consider aspects such as eligibility criteria, administrative processes, subsidy amounts, or other systemic barriers that may limit access.*

### Alternative delivery and funding

In areas where Out of School Hours Care (OSHC) services and Vacation care are unavailable, alternative delivery and funding models are needed to ensure all children can access structured summer programs.

#### Alternative summer program providers

**Survey question 13:**  In some areas, such as rural or regional communities and lower SES urban areas, Out of School Hours Care (OSHC) and Vacation Care facilities are unavailable. What alternative delivery models could be used to provide summer programming for children in these areas?

*(open text)*

*Think broadly about creative or innovative approaches that could address the unique challenges of these settings.*

#### Alternative funding models for these providers

**Survey question 14:** For areas where traditional OSHC and Vacation Care programs are not available, alternative funding models may be needed to make summer programs accessible for families living on low incomes. What funding mechanisms or subsidy structures could support participation in summer programs for these families?

*Consider any approaches that could ensure affordability and sustainability while addressing the needs of underserved communities.*

# General

**Survey question 15:**  Do you have any other comments or thoughts on summer programming in Australia?

*Feel free to share additional ideas, insights, or feedback, including aspects not covered in this survey.*

# Demographics

Please confirm which stakeholder group you represent:

**Please your primary stakeholder group (select one) *(logic answers for sub-options)***

1. I work in a school (e.g., teacher, principal, school-support officer)
2. I work for a program provider or advocacy body for the extended care setting
   1. Public sector
   2. Private sector
3. I work for a local, state or federal government body (e.g., department for education, department for health).
4. I am a researcher/academic
5. I am a parent/guardian of school-aged child

**In which region do you live/work? (select from list)**

- - Australia
  - United States of America
  - Other (specify)

## Thank You

Thank you for taking the time to participate in Round 2 of our Delphi study on structured summer programs for Australian children. Your insights and expertise are invaluable in shaping programs that can make a meaningful difference in the lives of children and families, particularly those facing disadvantage.

The results from this survey round will be sent out with the final survey in early March. We look forward to your continued participation.

Delphi Survey – Round 3

P1

# Introduction

Thank you for participating in the **third and final round** of our Delphi study on structured summer programs for Australian children.

In Round 1, stakeholders identified key barriers, facilitators, and priorities for these programs, focusing on their importance in supporting children’s physical, mental, and social-emotional wellbeing. Key findings are available here (link).

In Round 2, stakeholders identified program features, delivery and funding models for summer programs. Key findings are available here (link to report).

This round aims to clarify priority actions to enhance access to summer programming for families living on low incomes and refine the most viable model for a pilot program.

Your insights will help directly shape recommendations for policymakers and funding bodies to support sustainable programs and contribute to the design of a pilot program.

P2

# Program cost, funding and investment strategy

Programs are expensive to run, and stakeholders identified cost as the biggest barrier to access. Charging a fee for programs can enhance perceived value and improve attendance, but high costs make programs inaccessible for families living on low incomes.

### QUESTION 1:

**If we were to pilot a structured summer program for disadvantaged children, what would be the most feasible funding model?** *(Select up to two)*

1. Fully government-funded
2. New, simplified co-contribution model (government funding + family contributions based on income)
3. Expansion of existing subsidies (e.g., childcare subsidy with eligibility criteria relaxed)
4. Philanthropy and corporate sponsorship
5. Community-based fundraising or partnerships
6. Other (please specify): _______________

### QUESTION 2:

**What should be the maximum daily cost for low-income families to make the program affordable while ensuring sustainability?** *(Select one)*

- No charge
- $1–$5
- $6–$10
- $11–$15
- $16 - $20
- Other (please specify): _______________

*Note: the median annual income for a single parent family is approximately $40,000 AUD ($25,300 USD) which equates to $770 AUD ($490 USD) per week*

# Program structure and feasibility

Previous rounds identified multiple possible delivery models for structured summer programs. In this question, we are refining which models are most feasible to pilot based on infrastructure, staffing, and accessibility.

### QUESTION 3:

**In order to run a summer holiday pilot program, additional services will need to be provided in areas where existing vacation care are inadequate (i.e. either no services operating, or current services are operating at capacity with no available spaces).**

**Based on the findings so far, what do you believe are viable delivery models to pilot for a structured summer program?** (tick all that apply)

1. **Open more services in existing centres:** Expanding existing Outside-School-Hours-Care/Vacation Care (operated within existing OSHC/Vacation Care facilities)
2. **Open new services run by the community:** New programs operated by community-based groups delivered through community centres, schools, sports clubs, NGOs
3. **Open new services through community partnerships:** Independent providers/companies delivering new programs within existing community settings (e.g. schools, community centres)
4. Other (please specify): _______________

*Optional comments*

### QUESTION 4:

**More staff will be required in order to expand summer program offerings. Where could additional staff be recruited from? (tick all that apply)**

- Recruitment of pre-service teachers and childcare workers (who meet child safety requirements but may require additional training)
- Recruitment of qualified teachers on summer holidays (e.g., casual, non-contract teachers)
- Recruitment of university students with appropriate basic qualifications (first aid, Working with Children Check), similar to US and European models.
- Utilising local community sector staff (e.g., from non-profits, government agencies etc.) including social workers, youth workers, personal care givers, recreation officers.
- Other (please specify): _______________

*Optional comments*

# Program Delivery & Attendance Requirements

Programs need to balance family flexibility with ensuring that children receive sufficient engagement to benefit from participation. In previous rounds, there was no clear agreement on the minimum attendance required for meaningful impact.

### QUESTION 6:

**In Round 2, we saw mixed responses on the minimum attendance required for children to benefit. For a pilot program operating for the duration of a school day (9am to 3:30pm), what do you believe is the most appropriate attendance target? *(Select one)***

- 1 day/week
- 2 days/week
- 3 days/week
- 4 days/week
- 5 days/week
- Other (please specify): _______________

### QUESTION 7:

Respondents indicated that programs need to balance availability (operating 5 days per week) with flexible participation options to meet diverse family needs.

Applying the current Childcare Subsidy scheme, families are allocated a total number of hours of subsidized care per fortnight. Common business practices mean that children attending a half day (i.e., 8am – 12noon, 4 hours) can have their allocated hours applied to the full day operating hours of the service (e.g., 10 hours), reducing the number of remaining hours of care available to the family for that fortnight.

When children attend only a portion of the day, what is the most desirable charging practice?

1. Allow charging of sessional attendance (i.e. children can attend one or both sessions: 8am – 12 noon or 12noon – 4pm)
2. Charge one fee across the whole day (8am – 4pm), regardless of attendance duration

*Optional comments*

### QUESTION 8:

**Transport was identified as a barrier to attendance in previous rounds, but there was no consensus on whether it should be provided as part of the program. In your opinion, how should transport be addressed? (Select one)**

- Transport should be provided for all children who need it
- Transport should be offered only in areas where access is a major barrier
- Families should be responsible for arranging transport
- Other (please specify): _______________

# Scaling & System-Wide Change

**Key Barriers to scaling summer programming**

Expanding summer programs nationally will require overcoming logistical and funding challenges.

### QUESTION 9: (optional)

**What are the biggest barriers to scaling structured summer programs nationally? *(Rank 1-7)***

- Lack of sustainable government funding
- High operational costs for providers
- Staffing shortages
- Lack of suitable venues
- Lack of options/expertise in delivering structured, holiday programs
- Low family engagement/uptake
- Regulatory burden to operate under the National Quality Framework

Optional: other comments

### QUESTION 10:

**To increase government buy-in, what policy priorities should summer programming align with?** *(Select 4)*

- Long term return on investment through improved health outcomes (e.g., obesity prevention)
- Summer programs support physical activity and reduced screen time in children
- Summer programs support school engagement and better education outcomes in the following school year
- Summer programs provide positive ways for children to spend their time, reducing opportunities for less desirable behaviours that have community costs (e.g., delinquency, crimes like vandalism, policing)
- Summer programs promote social-emotional wellbeing and support child mental-health
- Improved wellbeing outcomes for parents/guardians
- Support for workforce participation and economic productivity. For example, addressing high cost of childcare that prevents some parents entering/staying in the workforce.
- Addressing social inequities by supporting vulnerable children
- Alignment with existing government initiatives (e.g., childcare, education, health policies)
- Benefits for local communities: improved services, economic development, opportunities for young people
- Public appeal (general desire to have summer programming) and voter-friendly policy

### OPTIONAL QUESTION 11:

**In your opinion, what partnerships or infrastructure could be leveraged to scale summer programs more efficiently? (Open text response)**

### OPTIONAL QUESTION 12:

**Do you have any additional insights on the feasibility, scalability, or funding of structured summer programs? (Open text response)**

# Conclusion

Thank you for participating in this project to understand summer holiday programming in Australia. Your insights have been incredibly helpful.

We will share the final results with you soon, along with next steps for this research.
